# Negative cooperativity underlies dynamic assembly of the Par complex regulators Cdc42 and Par-3

**DOI:** 10.1016/j.jbc.2022.102749

**Published:** 2022-11-25

**Authors:** Elizabeth Vargas, Kenneth E. Prehoda

**Affiliations:** Department of Chemistry and Biochemistry, Institute of Molecular Biology, Eugene, Oregon, USA

**Keywords:** cell polarity, protein interactions, membrane organization, PDZ domains, negative cooperativity, aPKC, atypical PKC, BR, basic region

## Abstract

The Par complex polarizes diverse animal cells through the concerted action of multiple regulators. Binding to the multi-PDZ domain containing protein Par-3 couples the complex to cortical flows that construct the Par membrane domain. Once localized properly, the complex is thought to transition from Par-3 to the Rho GTPase Cdc42 to activate the complex. While this transition is a critical step in Par-mediated polarity, little is known about how it occurs. Here, we used a biochemical reconstitution approach with purified, intact Par complex and qualitative binding assays and found that Par-3 and Cdc42 exhibit strong negative cooperativity for the Par complex. The energetic coupling arises from interactions between the second and third PDZ protein interaction domains of Par-3 and the aPKC Kinase-PBM (PDZ binding motif) that mediate the displacement of Cdc42 from the Par complex. Our results indicate that Par-3, Cdc42, Par-6, and aPKC are the minimal components that are sufficient for this transition to occur and that no external factors are required. Our findings provide the mechanistic framework for understanding a critical step in the regulation of Par complex polarization and activity.

The polarization of animal cells by the Par complex is a highly dynamic, multistep process that begins when actomyosin-generated cortical flows transport membrane-bound Par complex from cellular regions where it is catalytically inactive to a single cortical domain where it becomes activated (1, 2, 3, 4, 5, 6, 7, 8). The transition from an inactive to active complex is mediated by the formation of two distinct complexes: one bound to the multi-PDZ protein Par-3 (Bazooka; Baz in *Drosophila*) and a Rho GTPase Cdc42-bound complex. Par-3 has many reported interactions with both Par complex components, atypical PKC (aPKC) and Par-6, whereas Cdc42 has one well-defined binding site on Par-6 (Fig. 1*A*) (9, 10, 11, 12, 13, 14, 15, 16, 17, 18, 19). The transition between these two regulators precisely controls Par complex polarization and activity, with Par-3 coupling the Par complex to cortical flow while inhibiting aPKC activity and GTP-bound Cdc42 maintaining the Par complex at the cell cortex while stimulating aPKC activity (5, 20, 21). Despite the critical importance of the transition from Par-3 to Cdc42 in the mechanism of Par-mediated polarity, very little is known about how it occurs.

**Figure 1:**
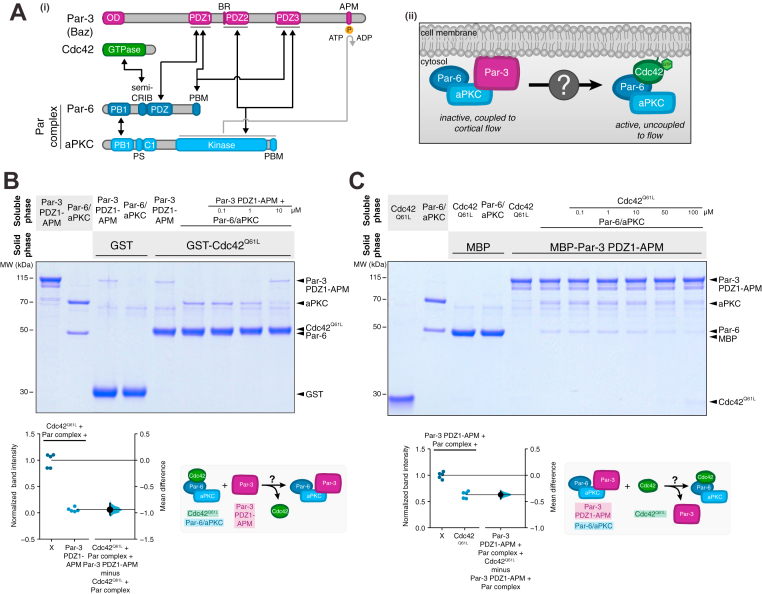
**Par-3 and Cdc42 bind to the Par complex with strong negative cooperativity.***A*, (i) domain architecture of Par-3, Cdc42, and the Par complex with reported interactions between Par-3, Cdc42, and the Par complex. *Black arrows* indicate reported interactions, and the *gray arrow* indicates phosphorylation. (ii) Schematic for the Par complex transition from Par-3 to Cdc42. *B*, effect of Par-3 PDZ1-APM on the interaction between Cdc42 and the Par complex. Solid-phase (glutathione resin)-bound glutathione-*S*-transferase (GST)–fused Cdc42Q61L (constitutively active Cdc42) incubated with Par complex and/or increasing concentrations of Par-3 PDZ1-APM. *Shaded regions* indicate the fraction applied to the gel (soluble-phase or solid-phase components after mixing with soluble-phase components and washing). Gardner–Altman estimation plot of normalized Cdc42-bound Par complex (Par-6 or aPKC) band intensity in the absence and presence of Par-3. The results of each replicate (*filled circles*) are plotted on the *left* and the mean difference is plotted on the *right* as a bootstrap sampling distribution (*shaded region*) with a 95% confidence interval (*black* error bar). *C*, effect of Cdc42 on the interaction between Par-3 and the Par complex. Solid-phase (amylose resin)–bound maltose-bound protein (MBP)-fused Par-3 PDZ1-APM incubated with Par complex and/or increasing concentrations of Cdc42Q61L. Labeling as described in (*B*). Gardner–Altman estimation plot of normalized Par-3-bound Par complex (Par-6 or aPKC) band intensity in the absence and presence of Cdc42Q61L. The results of each replicate (*filled circles*) are plotted on the *left* and the mean difference is plotted on the *right* as a bootstrap sampling distribution (*shaded region*) with a 95% confidence interval (*black* error bar).

While *in vivo* evidence indicates the Par complex switches from Par-3 to Cdc42-bound states, biochemical evidence suggests that Par-3 and Cdc42 can bind the Par complex simultaneously to form a quaternary complex. A coimmunoprecipitation experiment using cell extracts found that Cdc42-bound Par complexes also contain Par-3 (9). However, *in vivo* evidence indicate that there are two distinct cortical pools of Par complex, colocalizing with either Par-3 or Cdc42, and loss of Cdc42 increases the amount of Par-3-bound complex (3, 5, 20, 22). The reported ability of Cdc42 and Par-3 to bind simultaneously to the Par complex has influenced models for how the transition between the regulators could occur *in vivo*. In one model, Cdc42 briefly docks onto Par-3-bound Par complex and activates aPKC, resulting in the phosphorylation and release of Par-3 from the complex (23, 24, 25). However, recent studies show that phosphorylation of Par-3 by aPKC does not dissociate Par-3 from the Par complex (18, 26). In another proposed model, actomyosin contractility mechanically dissociates Par-3 clusters and facilitates the Par complex transition to Cdc42 (5, 20, 21).

Because the available biochemical data suggest that Par-3 and Cdc42 can bind simultaneously to the Par complex, models for the transition between the two regulators necessarily include other mechanisms (*e*.*g*., phosphorylation) or cellular components (*e*.*g*., actomyosin contractility). However, the limited *in vitro* evidence is based on results from cell extracts or experiments using truncated proteins. Additionally, the numerous reported interactions between Par-3 and the Par complex have made it challenging to understand how the Par-3-bound Par complex is regulated. Finally, very little structural information is known about the Par complex and whether the Par-3 and Cdc42 binding sites are in close proximity to one another to regulate the formation of these complexes. Here, we have used a biochemical reconstitution approach with purified components to determine the elements sufficient for Par complex switching between Par-3 and Cdc42. The results provide the mechanistic framework for understanding how the Par complex transitions from Par-3 to Cdc42 to form two distinct complexes.

## Results

### Par-3 and Cdc42 bind with negative cooperativity to the Par complex

Although Par-3 and Cdc42 are thought to form mutually exclusive complexes with the Par complex *in vivo*, they have been shown to bind simultaneously in a coimmunoprecipitation experiment using cell extracts (9). We examined whether Par-3 and Cdc42 influence one another’s binding to the Par complex using a reconstitution system. We performed a qualitative affinity chromatography (pull-down) assay with purified Par complex and Par-3 PDZ1-APM (a fragment containing all known interaction motifs between Par-3 and Par-6/aPKC) and GST-fused Cdc42^Q61L^ (constitutively active). The binding buffer included ATP to ensure that the aPKC kinase domain did not form a stalled complex with its phosphorylation site on Par-3.

We formed a complex of Cdc42-bound Par-6/aPKC by placing GST-Cdc42^Q61L^ on the solid phase and incubating with soluble, purified Par complex. We assessed the effect of Par-3 on the Cdc42-bound Par complex by adding increasing concentrations of Par-3 PDZ1-APM. If Par-3 binding to the Par complex had no effect on Cdc42 binding or the proteins bound with positive cooperativity, we expected that Par-3 would become part of the solid phase complex and the amount of Par complex adhered to the solid phase would stay the same or increase. Alternately, if the Cdc42 and Par-3 binding sites exhibited negative cooperativity, either *via* direct steric occlusion or an allosteric mechanism, little or no Par-3 would be part of the Cdc42-bound solid phase complex, and the amount of Par complex on the solid phase would decrease (as the affinity of the Par complex for Cdc42 was reduced by binding to Par-3). We observed that addition of Par-3 significantly reduced the amount of Par complex associated with solid phase Cdc42^Q61L^ (Fig. 1*B*). Furthermore, little or no additional Par-3 appeared in the solid phase relative to a GST control. Our results indicate that in the context of these four proteins, Cdc42 and Par-3 bind with negative cooperativity to Par-6/aPKC.

In a system with two distinct binding sites coupled to one another *via* negative cooperativity, each protein should reduce the affinity of the Par complex for the other. However, the effect of Cdc42 on Par-3 binding to the Par complex is complicated by the many potential Par-3-binding sites on the Par complex (Fig. 1*A*). In principle, not all Par-3-binding sites could be coupled to Cdc42 binding, a scenario in which addition of Cdc42 to solid phase Par-3-bound Par complex might not significantly alter the amount of solid phase Par complex. To determine if Cdc42 influences Par-3-bound Par complex, we adsorbed Par complex bound to MBP-Par-3 PDZ1-APM to the solid phase and examined the effect of increasing concentrations of Cdc42^Q61L^. We observed that addition of Cdc42 reduced the amount of Par complex associated with solid phase Par-3 and Cdc42 was not significantly incorporated into the solid phase (Fig. 1*C*). Displacement of Par complex from Par-3 required a significantly higher concentration of Cdc42 than we observed for Par-3 displacement of Cdc42-bound complex.

Our results indicate that Par-3 and Cdc42 compete for binding to the Par complex (*i*.*e*., negative cooperativity) and that a quaternary complex does not form at levels detectable in our assay. Our results may differ from previous studies using cell extracts because aPKC’s kinase domain is known to form stalled complexes with substrates like Par-3 when ATP is not available to complete the catalytic cycle (forming a persistent interaction rather than a transient interaction) (18, 26). Additionally, other cellular factors could potentially allow Par-3 and Cdc42 to bind to the Par complex simultaneously. In terms of understanding how the Par complex might transition from Par-3 to Cdc42, our results demonstrate that no other proteins are required—Par-3 and Cdc42 alone are sufficient to form mutually exclusive complexes with the Par complex.

### Par-3 may bind the Par complex with higher affinity than Cdc42

**Figure 2:**
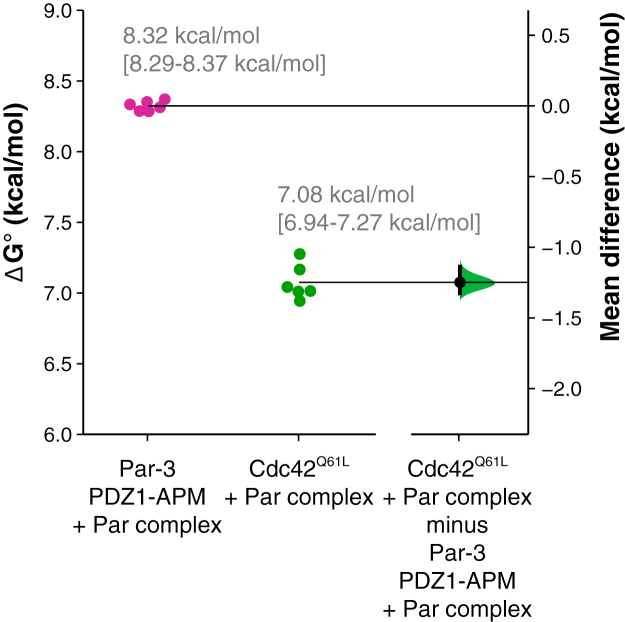
**Par-3 binds to the Par complex with a greater affinity than Cdc42 in a supernatant depletion assay.** Gardner–Altman estimation plot of Par-3 PDZ1-APM/Par complex and Cdc42Q61L/Par complex binding affinities measured using a supernatant depletion assay. The results of each replicate (*filled circles*) are plotted on the *left* and the mean difference is plotted on the *right* as a bootstrap sampling distribution (*shaded region*) with a 95% confidence interval (*black* error bar).

Our results indicate that Par-3 is more effective at displacing Cdc42 from the Par complex than Par-3 is at displacing Cdc42. The asymmetry in Par complex displacement could be explained by a higher affinity of Par-3 for the Par complex compared to Cdc42. The affinity of Par-3 PDZ1-APM for the Par complex is known (19), but while Cdc42’s affinity for the Par-6 CRIB-PDZ fragment has been reported (13), its affinity for the full Par complex has been unknown. To understand why Par-3 is more effective at displacing Cdc42 from the Par complex, we measured binding affinities for the Par complex using a supernatant depletion assay (27). Similar to a previous report using the same assay (19), we found that Par-3 PDZ1-APM binds the Par complex with high affinity (Fig. 2; *K*_*D*_ of 0.6 μM or ΔG° of 8.3 kcal/mol). We measured a substantially weaker affinity of Cdc42 (using the Q61L constitutively active variant) for the Par complex (Fig. 2; *K*_*D*_ of 5.4 μM or ΔG° of 7.1 kcal/mol). This affinity is significantly lower than a previous report of 0.05 μM for Cdc42 binding to the Par-6 CRIB-PDZ fragment using a FRET-based assay (13). To determine if the source of the difference is Cdc42 binding to a Par-6 fragment *versus* the full Par complex, we measured the Cdc42 interaction with Par-6 CRIB-PDZ with the supernatant depletion (Fig. S1). While the resulting affinity of 2.3 μM is slightly higher than that for the full Par complex, it remains substantially weaker than the FRET-based value (which is a higher affinity than the Par-3 interaction with the Par complex). We are unsure of the source of this discrepancy, and while our results suggest that Par-3 displaces Cdc42 from the Par complex more efficiently because of an intrinsic difference in affinity, it is possible that there is another source for this phenomenon.

### The Par-3 PDZ2–aPKC Kinase-PBM interaction mediates the displacement of Cdc42 from the Par complex

**Figure 3:**
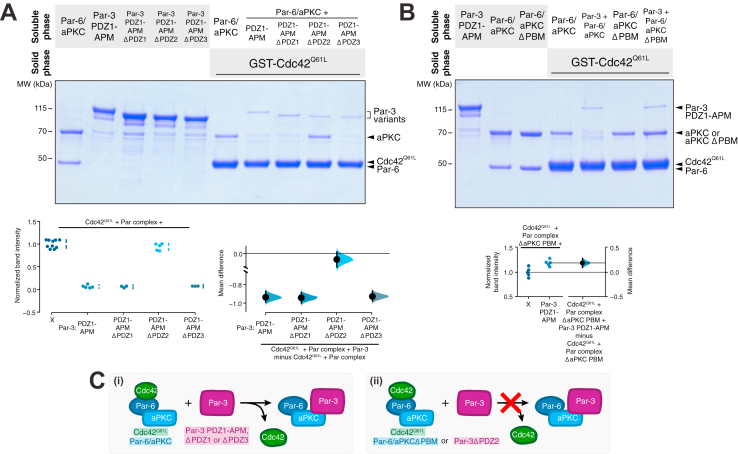
**The Par-3 PDZ2 interaction with the aPKC PBM is required to displace Cdc42 from the Par complex.***A*, effect of removing individual Par-3 PDZ domains in the context of PDZ1-APM on the displacement of Cdc42 from the Par complex. Solid-phase (glutathione resin)–bound glutathione-*S*-transferase (GST)–fused Cdc42Q61L incubated with Par complex and/or Par-3 PDZ1-APM, ΔPDZ1, ΔPDZ2, or ΔPDZ3. *Shaded regions* indicate the fraction applied to the gel (soluble-phase or solid-phase components after mixing with soluble-phase components and washing). Cumming estimation plot of normalized Cdc42-bound Par complex (Par-6 or aPKC) band intensity in the absence and presence of Par-3 variants. The result of each replicate (*filled circles*) along with the mean and SD (gap and bars next to *circles*) are plotted on the *left* and the mean differences are plotted on the *right* as a bootstrap sampling distribution (*shaded region*) with a 95% confidence interval (*black* error bar). Replicates not included in the plot (<5 *circles*) had a band intensity that was not detectable. *B*, effect of removing the aPKC PBM in the context of the intact Par complex on Par-3’s ability to displace Cdc42 from the Par complex. GST-fused Cdc42Q61L incubated with Par complex or Par complex ΔaPKC PBM and/or Par-3 PDZ1-APM. Labeling as described in (*A*). Gardner–Altman estimation plot of normalized Cdc42-bound Par complex ΔaPKC PBM (Par-6 or aPKC) band intensity in the absence and presence of Par-3. The results of each replicate (*filled circles*) are plotted on the *left* and the mean difference is plotted on the *right* as a bootstrap sampling distribution (*shaded region*) with a 95% confidence interval (*black* error bar). *C*, summary of Cdc42, Par-3, and Par-6/aPKC elements that are necessary for negative cooperativity between Par-3 and Cdc42 for the Par complex.

Given that Par-3 and Cdc42 bind with negative cooperativity to Par-6/aPKC, we sought to identify the binding sites on the Par complex that are coupled. While the interaction between Cdc42 and Par-6 semi-CRIB is well established, several interactions between Par-3 and the Par complex have been identified (Fig. 1*A*) (9, 10, 11, 12, 13, 14, 15, 16, 17, 18, 19). We excluded the interaction of the aPKC kinase domain with its phosphorylation site on Par-3 (*i*.*e*., the Par-3 APM) because the interaction is transient in the presence of ATP, as expected for an enzyme–substrate interaction (18, 26). Given that each Par-3 PDZ domain reportedly interacts with either Par-6 or aPKC, more than one interaction between Par-3 and the Par complex could be involved in displacement of Cdc42 from the Par complex. However, if only one of the interactions between Par-3 and the Par complex displaces Cdc42 from the Par complex, deletion of the required Par-3 element would eliminate Par-3’s negative cooperativity with Cdc42 for the Par complex. Alternately, removal of more than one Par-3 element might be necessary to eliminate displacement of Cdc42 from the Par complex by Par-3. To distinguish between these possibilities, we generated deletions of individual Par-3 PDZ domains in the context of the PDZ1-APM fragment and tested which Par-3 elements are involved in displacing Cdc42 from the Par complex. We examined the effect of Par-3 PDZ1-APM, ΔPDZ1, ΔPDZ2, or ΔPDZ3 on Cdc42-bound Par complex. We did not detect an effect of removing PDZ1 or PDZ3 on Par-3’s ability to displace Cdc42 from the Par complex (Fig. 3*A*). In contrast, deletion of Par-3 PDZ2 eliminated displacement of Cdc42 such that the amount of Par complex associated with solid phase Cdc42 did not change upon addition of Par-3 PDZ1-APM ΔPDZ2 (Fig. 3*A*). Our results indicate that neither Par-3 PDZ1 nor PDZ3 are required for negative cooperativity with Cdc42 for the Par complex and that displacement of Cdc42 from the Par complex by Par-3 is dependent on the PDZ2 domain.

We recently discovered that Par-3 PDZ2 and PDZ3 interact with aPKC Kinase Domain-PBM (KD-PBM) module (19). Given that Par-3 PDZ2 is required to displace Cdc42 from the Par complex, we examined whether the aPKC PBM was also required for this activity. We found that removing the aPKC PBM (Par-6/aPKC ΔPBM) prevented Par-3 from displacing Cdc42 from the Par complex (Fig. 3*B*). Our results indicate that Par-3 PDZ2 and aPKC PBM are necessary for Par-3 to disrupt the Cdc42–Par complex interaction (Fig. 3*C*).

### Par-3 BR-PDZ2 and PDZ3 displace Cdc42 from the Par complex

**Figure 4:**
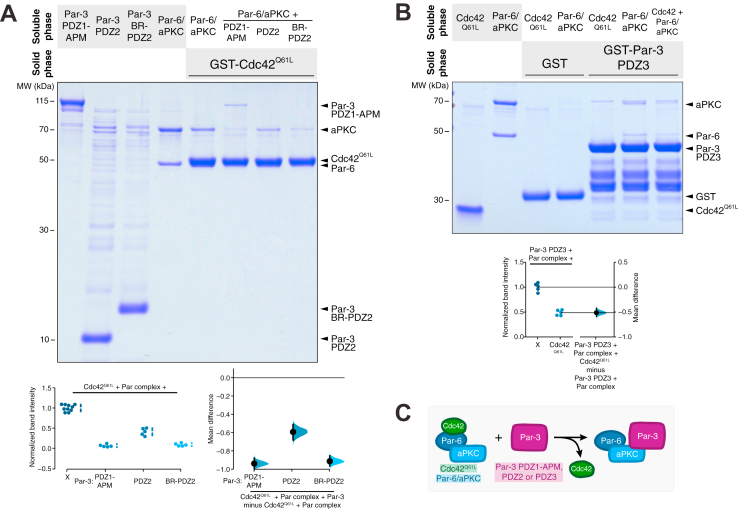
**Par-3 PDZ2 and PDZ3 are sufficient for displacement of Cdc42 from the Par complex.***A*, effect of Par-3 elements on the interaction between Cdc42 and the Par complex. Solid-phase (glutathione resin)–bound glutathione-*S*-transferase (GST)–fused Cdc42Q61L incubated with Par complex and/or Par-3 PDZ1-APM, PDZ2, or BR-PDZ2. *Shaded regions* indicate the fraction applied to the gel (soluble-phase or solid-phase components after mixing with soluble-phase components and washing). Cumming estimation plot of normalized Cdc42-bound Par complex (Par-6 or aPKC) band intensity in the absence and presence of Par-3 variants. The result of each replicate (*filled circles*) along with the mean and SD (gap and bars next to *circles*) are plotted on the *left* and the mean differences are plotted on the *right* as a bootstrap sampling distribution (*shaded region*) with a 95% confidence interval (*black* error bar). *B*, effect of Cdc42 on the interaction between Par-3 PDZ3 and the Par complex. GST-fused Par-3 PDZ3 incubated with Par complex and/or Cdc42Q61L. Labeling as described in (*A*). Gardner–Altman estimation plot of normalized Par-3-bound Par complex (Par-6 or aPKC) band intensity in the absence and presence of Cdc42Q61L. The results of each replicate (*filled circles*) are plotted on the *left* and the mean difference is plotted on the *right* as a bootstrap sampling distribution (*shaded region*) with a 95% confidence interval (*black* error bar). *C*, summary of Cdc42, Par-3, and Par-6/aPKC elements that are sufficient for negative cooperativity between Par-3 and Cdc42 for the Par complex.

Given the requirement of the aPKC KD-PBM for Par-3’s ability to displace Cdc42 from the Par complex, we examined whether the Par-3 domains that bind the KD-PBM (PDZ2 and PDZ3) were each sufficient for this activity. We recently discovered a conserved basic region (BR) at the N-terminal end of Par-3 PDZ2 that increases PDZ2’s affinity for the Par complex, so we also examined the effect of Par-3 BR-PDZ2 (19). We found that PDZ2 alone was sufficient to displace Cdc42 from the Par complex but was not as effective as PDZ1-APM such that some Par complex remained bound to solid phase Cdc42 (Fig. 4*A*). In contrast, BR-PDZ2 displaced Cdc42 to a similar extent as PDZ1-APM and resulted in little to no Par complex associated with solid phase Cdc42 (Fig. 4*A*). We conclude that Par-3 BR-PDZ2 can sufficiently displace Cdc42 from the Par complex.

**Figure 5:**
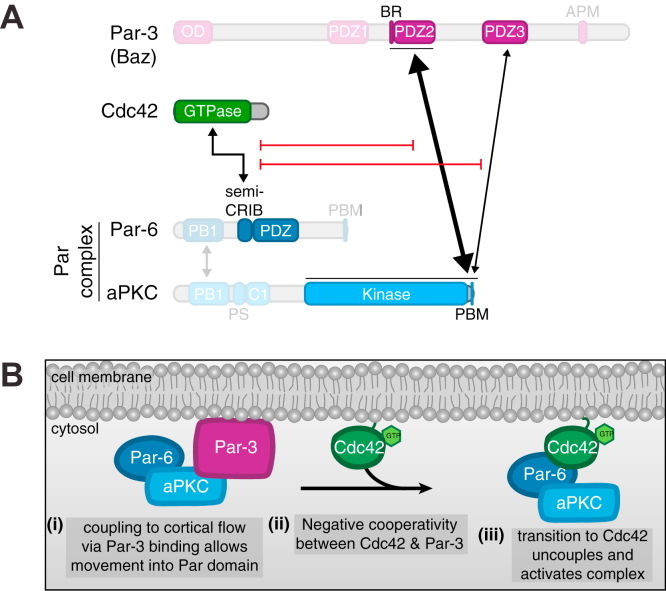
**Model for transition from Par-3- to Cdc42-bound Par complex.***A*, domain architecture demonstrating the interactions between Par-3, Cdc42, and the Par complex that are sufficient for regulating the transition between a Par-3- and Cdc42-bound Par complex. *B*, model for polarization of the Par complex through the formation of distinct Par-3- and Cdc42-bound complexes. (i) Par-3-bound Par complex is coupled to cortical flow, allowing it to move toward the Par domain, (ii) active Cdc42 binds to Par-6 and inhibits the Par-3 BR-PDZ2/PDZ3–aPKC kinase-PBM interaction, resulting in the displacement of Par-3 from the Par complex and the formation of a Cdc42-bound Par complex, and (iii) Cdc42-bound Par complex is polarized and active. BR, basic region.

Like Par-3 PDZ2, Par-3 PDZ3 was found to interact with the Par complex utilizing a similar binding mode. Thus, we also tested the ability of Par-3 PDZ3 to displace Cdc42 from the Par complex. Given that Par-3 PDZ3 has a weak binding affinity for the Par complex (*K*_*D*_ of 78.9 μM) (19), we were unable to detect any significant change in the amount of Par complex bound to solid phase Cdc42 (data not shown). Therefore, we instead formed a Par-3 PDZ3-bound Par complex utilizing GST-Par-3 PDZ3 on the solid phase and soluble Par complex and examined the effect of Cdc42^Q61L^. We observed that addition of Cdc42 resulted in a reduction in the amount of Par complex bound to solid phase Par-3 PDZ3, indicating that Cdc42 is sufficient to displace PDZ3 from the Par complex (Fig. 4*B*). Thus, our finding that Par-3 PDZ1-APM ΔPDZ2 cannot displace Cdc42 (Fig. 3*A*) likely arises from the low binding affinity of PDZ3 for the Par complex compared to BR-PDZ2. Altogether, our results indicate that the Par-3 BR-PDZ2 and PDZ3–aPKC kinase domain-PBM (predominantly through BR-PDZ2) interactions negatively cooperate with the Cdc42–Par-6 CRIB-PDZ interaction (Figs. 4*C* and 5*A*).

## Discussion

We examined how the Par complex transitions from Par-3- to Cdc42-bound states, a step that is thought to be critical for forming the Par cortical domain. In this model, the large size of oligomerized Par-3 couples the Par complex to actomyosin-driven directional cortical flows that transport the complex to its active domain (5, 20, 21). Once in this membrane region, the complex dissociates from Par-3 and binds Cdc42, allowing it to remain on the cortex and become activated. While the transition from Par-3 to Cdc42 is central to this model, little has been known about how switching between binding of each regulator occurs. We used a biochemical reconstitution approach to understand the mechanism of regulator switching, with the goal of identifying the minimal set of components required for the transition. A previous study that used coimmunoprecipitation from cultured cell extracts concluded that Cdc42 and Par-3 can bind simultaneously to the Par complex, suggesting that other cellular components are required (9). For example, mechanical separation of the complexes by actomyosin-generated contraction has been proposed as one possibility (5, 20, 21). Using purified components, we found that external factors are not required as Cdc42 and Par-3 bind to the Par complex with strong negative cooperativity (Fig. 5*B*). In this section, we examine the implications of our findings and speculate on key outstanding issues that remain in understanding this critical step in Par-mediated polarity.

How can the strong negative cooperativity between Cdc42 and Par-3 binding to the Par complex that we observed be reconciled with the previous observation of a quaternary complex? There are several possible explanations. First, it does not appear that ATP was included in the previous binding experiment, perhaps because it was not clear that Par-3 is an aPKC substrate at the time. We have found that the phosphorylation site on Par-3 can form a stalled complex with the aPKC kinase domain when ATP is not present (18, 26). Alternately, since the experiment was performed in extracts, it's possible that additional factors were present that inhibit negative cooperativity, allowing Cdc42 and Par-3 to bind simultaneously. Finally, the presence of negative cooperativity does not necessarily preclude formation of a quaternary complex, albeit at reduced levels.

The nature of the Par complex interaction with Par-3 has been enigmatic because many distinct interactions have been reported (9, 10, 11, 12, 13, 14, 15, 16, 17, 18, 19). We examined which Par-3 interactions are coupled to Cdc42 binding and found that only one involving the aPKC kinase domain and PDZ-binding motif (KD-PBM) is affected by Cdc42. This site binds with highest affinity to Par-3's BR-PDZ2 domain but with weaker affinity to PDZ3 (19). Our results are consistent with the reported relative affinities—BR-PDZ2 most effectively reduces Cdc42 binding to the Par complex. We found that binding to the aPKC KD-PBM is both necessary and sufficient to displace Cdc42 and that Cdc42 can nearly completely displace Par-3 PDZ1-APM from the Par complex. These results indicate that the other reported interactions of Par-3 with the Par complex are not likely to be relevant to this step of Par-mediated cell polarity.

A central consequence of our findings for Par complex function is that the transition from Par-3-bound to Cdc42-bound Par complex does not require additional factors. Thus, the presence of Cdc42 alone could be sufficient for the transition to take place. However, our results do not preclude the possibility that other factors assist in complex switching. As we found that the affinity of Cdc42 for the Par complex may be lower than that of Par-3, a higher concentration of Cdc42 could be required to achieve the same amount of Cdc42-bound complex. It is possible that the amount of Cdc42-bound complex does not need to be in excess of Par-3-bound complex or that the concentration of active Cdc42 is higher than Par-3. Alternately, other factors could influence the affinity of Cdc42 with the Par complex—ligands of the Par-6 PDZ domain have been found to be coupled to Cdc42 binding, for example (28, 29).

How might the Cdc42- and Par-3-binding sites be coupled? The strongest negative cooperativity arises from a steric mechanism, where the binding sites would require steric overlap for Cdc42 and Par-3 to bind simultaneously. Alternately, in an allosteric mechanism, binding is coupled to changes in structure or dynamics that reduce the affinity for the other regulator. While the binding site for Cdc42 is on Par-6 (semi-CRIB) and Par-3's binding site is on aPKC (KD-PBM), little is known about the structural arrangement of the domains within the Par complex. Thus, it is formally possible that the semi-CRIB and KD-PBM are near one another, and it has been speculated that the PDZ domain adjacent to the semi-CRIB interacts with the KD-PBM (30). However, it is also clear that the Par complex is highly allosteric, as aPKC is autoinhibited from an intramolecular interaction between its pseudosubstrate and the kinase domain and that Par-6 partially disrupts this interaction (31). Additional biochemical and structural information will be required to uncover the mechanism of energetic coupling between Cdc42 and Par-3 binding to the Par complex.

## Experimental procedures

### Protein expression

#### Bacterial cells

Plasmids were transformed into BL21-DE3 cells, aliquoted onto LB + AMP plates, and grown at 37 °C for 18 h. Colonies were picked to inoculate 100 ml LB + AMP starter cultures and grown at 37 °C for 2 to 3 h until an *A*_600_ of 0.4 to 1.0 was reached. Starter cultures were then diluted into 2 l LB + AMP, grown at 37 °C to an *A*_600_ of 0.8 to 1.0, and induced with 500 μM IPTG for 3 h. Cultures were centrifuged at 5000 rpm for 15 to 20 min and pellets were resuspended in nickel lysis buffer (50 mM NaH_3_PO_4_, 300 mM NaCl, 10 mM Imidazole, pH 8.0), GST lysis buffer (IX PBS, 1 mM DTT, pH 7.5), or maltose lysis buffer (20 mM Tris, 200 mM NaCl, 1 mM EDTA, 1 mM DTT, pH 7.5). Resuspended pellets were then frozen in liquid N_2_ and stored at −80 °C.

#### Mammalian cells

Par-6 and aPKC plasmids were coexpressed in FreeStyle 293-F cells as previously described (18, 19, 31). Briefly, cells were grown in FreeStyle 293 expression media in shaker flasks at 37 °C with 8% CO_2_ and transfected with either 293fectin or ExpiFectamine (see Manufacturer’s protocol for more details). After 48 h, cells were centrifuged 1-2x at 500*g* for 3 min and cell pellets were resuspended in nickel lysis buffer (50 mM NaH_3_PO_4_, 300 mM NaCl, 10 mM Imidazole, pH 8.0). Resuspended pellets were then frozen in liquid N_2_ and stored at −80 °C.

### Protein purification

#### Bacterial cells

Resuspended pellets were thawed and then lysed by probe sonication at 70% amplitude, 0.3 s/0.7 s on/off pulse rate, 3× 1 min. To pellet cellular debris, lysates were centrifuged at 15,000 rpm for 20 min. Lysates for GST-Cdc42^Q61L^ and GST-Par-3 PDZ3 were aliquoted, frozen in liquid N_2_, and stored at −80 °C. For all other proteins, lysates were incubated with resin (amylose for most MBP-fused proteins and cobalt or nickel resin for His-fused proteins; MBP-Par-3 PDZ1-APM-His was His-purified) for 30 to 60 min at 4 °C with mixing. Protein-bound resin was washed 3× with nickel lysis buffer (50 mM NaH_3_PO_4_, 300 mM NaCl, 10 mM Imidazole, pH 8.0) or maltose lysis buffer (20 mM Tris, 200 mM NaCl, 1 mM EDTA, 1 mM DTT, pH 7.5) and then eluted in 0.5 to 1.8 ml fractions with nickel elution buffer (50 mM NH_3_PO_4_, 300 mM NaCl, 300 mM imidazole, pH 8.0) or maltose elution buffer (maltose lysis buffer, 10 mM maltose). Protein-containing fractions were pooled and buffer exchanged into 20 mM Hepes, pH 7.5, 100 mM NaCl, and 1 mM DTT using a PD10 desalting column. Protein was then concentrated using a Vivaspin 20 centrifugal concentrator, aliquoted, frozen in liquid N_2_, and stored at −80 °C.

#### Mammalian cells

Resuspended 293F pellets were thawed, lysed by probe sonication at 70% amplitude, 0.3 s/0.7 s on/off pulse rate, 4× 1 min, and centrifuged at 15,000 rpm for 20 min. Lysates were incubated with resin (cobalt or nickel resin) for 30 to 60 min at 4 °C with mixing. Protein-bound resin was washed 3× with nickel lysis buffer (50 mM NaH_3_PO_4_, 300 mM NaCl, 10 mM imidazole, pH 8.0), with the first and second washes containing 100 μM ATP and 5 mM MgCl_2_. Protein was eluted in 0.5 to 0.6 ml fractions with nickel elution buffer (50 mM NH_3_PO_4_, 300 mM NaCl, 300 mM imidazole, pH 8.0) and protein-containing fractions were then pooled together. Protein was buffer exchanged into 20 mM Tris, pH 7.5, 100 mM NaCl, 1 mM DTT, 100 μM ATP, and 5 mM MgCl_2_ using a PD10 desalting column and then purified by anion-exchange chromatography using an AKTA FPLC protein purification system. Protein was filtered, injected into a Source Q column, and eluted with a salt gradient of 100 to 500 mM NaCl. Par complex–containing fractions were pooled and buffer exchanged into 20 mM Hepes, pH 7.5, 100 mM NaCl, 1 mM DTT, 100 μM ATP, and 5 mM MgCl_2_ using a PD10 desalting column. Protein was then concentrated using a Vivaspin 20 centrifugal concentrator, aliquoted, frozen in liquid N_2_, and stored at −80 °C.

### Qualitative binding assay (affinity chromatography)

Bacterial lysates were incubated with resin (glutathione or amylose) for 30 min at 4 °C and washed 4× with binding buffer (20 mM Hepes pH 7.5, 100 mM NaCl, 5 mM MgCl_2_, 0.5% Tween-20, 1 mM DTT, and 200 μM ATP). Soluble proteins were added to protein-labeled resin and incubated at room temperature (RT) with rotational mixing (incubation times of 10 min for MBP-Par-3 PDZ1-APM and 60 min for GST-fused proteins). Resin was washed 2-3× with binding buffer and proteins were eluted with 4X LDS sample buffer. Samples were run on a 12% Bis–Tris gel and stained with Coomassie Brilliant Blue R-250. Band intensities were quantified using ImageJ (v1.53a; https://imagej.net). The normalized band intensity was determined by first obtaining the mean of Par complex (either Par-6 or aPKC) band intensities (in the presence of no other soluble protein) and then dividing each band intensity value by that mean. All data were analyzed using Microsoft Excel (v16.53), GraphPad Prism (v9.2) (GraphPad Software Inc), and DABEST (32).

### Quantitative binding assay (supernatant depletion)

Bacterial lysates were incubated with resin (GSH or amylose) for 30 min at 4 °C and washed 6× (3× quick washes, 3× 5 min washes) with binding buffer (20 mM Hepes pH 7.5, 100 mM NaCl, 5 mM MgCl_2_, 0.5% Tween-20, 1 mM DTT, and 200 μM ATP). Two-fold serial dilutions of protein-bound resin were prepared with unlabeled resin as previously described (18, 19). Soluble protein (“receptor” or R) was added to protein-bound resin (“ligand” or L) and incubated at RT with rotational mixing (incubation times of 10 min for MBP-Par-3 PDZ1-APM and 60 min for GST-Cdc42^Q61L^). Samples containing only unlabeled resin and soluble protein were used as a negative control for binding. After incubation, samples were centrifuged and an aliquot of the supernatant was collected and diluted in 4X LDS sample buffer. Samples were then run on a 12% Bis–Tris gel and stained with Coomassie Brilliant Blue R-250. Solid phase protein concentration was verified using a standard curve generated with known concentrations of a protein standard. All band intensities were quantified using ImageJ (v1.53a). The fraction of soluble phase protein (R) bound to solid phase protein (L) at a specific concentration of L ([L] = x) was determined with the following equation:

$${Fraction\;bound\;\left(F_{b}\right)}_{\left[L\right]=x}=1-\frac{{R\;band\;intensity}_{\left[L\right]=x}}{{R\;band\;intensity}_{\left[L\right]=0}}$$

We then determined the dilution at which L resulted in 30% to 60% depletion (F_b_ = 0.3–0.6) and repeated the assay at this dilution in sextuplicate. Using F_b_, we determined the binding equilibrium dissociation constant (*K*_*D*_) with the following equation:

$$K_{d}=\frac{\left[L\right]\left[R\right]}{\left[LR\right]}$$

where [L] and [R] are the concentrations of free L and R at equilibrium and [LR] is the concentration of L bound to R.

$$K_{d}=\frac{\left({\left[L\right]}_{total}-\left[LR\right]\right)\left({\left[R\right]}_{total}-\left[LR\right]\right)}{\left[LR\right]}$$

$$K_{d}=\frac{\left({\left[L\right]}_{total}-F_{b}{\left[R\right]}_{total}\right)\left({\left[R\right]}_{total}-F_{b}{\left[R\right]}_{total}\right)}{F_{b}{\left[R\right]}_{total}}$$

We also used the equation for the standard Gibbs free energy exchange to determine the binding energy of these interactions:

$$\Delta G{}^{\circ}=-RT\;\ln\left[K_{d}\right]$$

All data were analyzed using Microsoft Excel (v16.53), GraphPad Prism (v9.2), and DABEST (32).

## Key resources table

**Table:** 

Reagent or resource type	Reagent or resource	Source	Identifier	Additional information
Recombinant protein	MBP-Par-3 PDZ1-APM	PMID: 32084408		Expressed in BL21 cells from pMAL Par-3309–987-His; His-purified; *Drosophila melanogaster* protein
Recombinant protein	MBP-Par-3 PDZ1-APM ΔPDZ1	This paper		Cloned by Q5 site-directed mutagenesis; expressed in BL21 cells from pMAL Par-3393–987-His; His-purified; *Drosophila melanogaster* protein
Recombinant protein	MBP-Par-3 PDZ1-APM ΔPDZ2	PMID: 35787373		Expressed in BL21 cells from pMAL Par-3309–987 Δ437–533-His; His-purified; *Drosophila melanogaster* protein
Recombinant protein	MBP-Par-3 PDZ1-APM ΔPDZ3	This paper		Cloned by Q5 site-directed mutagenesis; expressed in BL21 cells from pMAL Par-3309–987 Δ616–741-His; His-purified; *Drosophila melanogaster* protein
Recombinant protein	His-Par-3 PDZ2	PMID: 35787373		Expressed in BL21 cells from pET19 Par-3444–533; His-purified; *Drosophila melanogaster* protein
Recombinant protein	His-Par-3 BR-PDZ2	PMID: 35787373		Expressed in BL21 cells from pET19 Par-3426–533; His-purified; *Drosophila melanogaster* protein
Recombinant protein	GST-Par-3 PDZ3	PMID: 35787373		Expressed in BL21 cells from pGEX Par-3616–741; *Drosophila melanogaster* protein
Recombinant protein	His-Cdc42^Q61L^	This paper		Cloned by traditional methods; expressed in BL21 cells from pBH Cdc42 1–191 Q61L; His-purified; *Drosophila melanogaster* protein
Recombinant protein	GST-Cdc42^Q61L^	This paper		Cloned by traditional methods; expressed in BL21 cells from pGEX Cdc42 1–191 Q61L; *Drosophila melanogaster* protein
Recombinant protein	Par complex	PMID: 32084408		Expressed in 293F cells from pCMV His Par-6 1–351 and pCMV aPKC 1–606; His-purified; *Drosophila melanogaster* proteins
Recombinant protein	Par complex ΔaPKC PBM	PMID: 32084408		Expressed in 293F cells from pCMV His Par-6 1–351 and pCMV aPKC 1–600; His-purified; *Drosophila melanogaster* proteins
Recombinant protein	GST-Par-6 CRIB-PDZ	This paper		Cloned by Gibson assembly; expressed in BL21 cells from pGEX Par-6 130–256; *Drosophila melanogaster* protein
Recombinant protein	His-Par-6 CRIB-PDZ	This paper		Cloned by traditional methods; expressed in BL21 cells from pBH Par-6 130–255; His-purified; *Drosophila melanogaster* protein
Recombinant DNA	pCMV (mammalian expression plasmid)	ThermoFisher	10586014	
Recombinant DNA	pMAL c4X (bacterial expression plasmid)	Addgene	75288	
Recombinant DNA	pGEX 4T1 (bacterial expression plasmid)	Amersham	27458001	
Recombinant DNA	pBH (bacterial expression plasmid)	PMID: 15023337		
Recombinant DNA	pET19 (bacterial expression plasmid)	Millipore Sigma (Novagen)	69677	
Bacterial strain	TG1			Molecular cloning
Bacterial strain	BL21-DE3			Protein expression
Cell Line	FreeStyle 293-F	ThermoFisher	R79007	
Chemical	293fectin transfection reagent	ThermoFisher	12347019	
Chemical	ExpiFectamine 293 transfection kit	ThermoFisher	A14524	
Chemical	Freestyle 293 expression medium	ThermoFisher	12338018	
Chemical	Expi293 expression medium	ThermoFisher	A1435101	
Chemical	Opti-MEM	ThermoFisher	3198588	
Chemical	HisPur Cobalt resin	ThermoFisher	89965	
Chemical	HisPur NiNTA resin	ThermoFisher	88222	
Chemical	Amylose resin	NEB	E8021L	
Chemical	GSH resin	GoldBio	G250-100	
Chemical	Source 30Q anion exchange resin	GE Healthcare	17-1275-01	
Chemical	4× BOLT LDS sample buffer	ThermoFisher	B0007	
Chemical	PageRuler Plus prestained protein ladder, 10–250 kDa	ThermoFisher	26619	
Chemical	20× BOLT Mes SDS running buffer	ThermoFisher	B0002	
Chemical	Coomassie Brilliant Blue R-250	GoldBio	C-461-5	
Chemical	Q5 site-directed mutagenesis kit	NEB	E0552S	
Chemical	Gibson assembly cloning kit	NEB	E5510S	
Other	125 ml Erlenmeyer Flasks	VWR	89095-258	
Other	250 ml Erlenmeyer Flasks	VWR	89095-266	
Other	Bolt 12% Bis–Tris gels	ThermoFisher	NW00125BOX	
Other	PD-10 desalting columns	VWR	95017-001	
Other	VivaSpin 20 MWCO 5 kDa	Cytiva	28932359	
Other	VivaSpin 20 MWCO 10 kDa	Cytiva	28932360	
Other	VivaSpin 20 MWCO 30 kDa	Cytiva	28932361	
Software	ImageJ	NIH		https://imagej.nih.gov/ij/
Software	Prism	GraphPad Software		https://www.graphpad.com/
Software	Estimation statistics BETA	PMID: 31217592		www.estimationstats.com

## Data availability

Example SDS-PAGE data are available within the article. Other gels used for additional quantification are available on request to K. E. P.

## Supporting information

This article contains supporting information.

## Conflict of interest

The authors declare that they have no conflicts of interest with the contents of this article.
