# Supplementary material for: Negative cooperativity underlies dynamic assembly of the Par complex regulators Cdc42 and Par-3
Source: J Biol Chem. 2022 Nov 25;299(1):102749. doi: 10.1016/j.jbc.2022.102749 (PMC9793311; doi:10.1016/j.jbc.2022.102749)
Supplement: Supplemental Figure S1 [file mmc1.pdf]

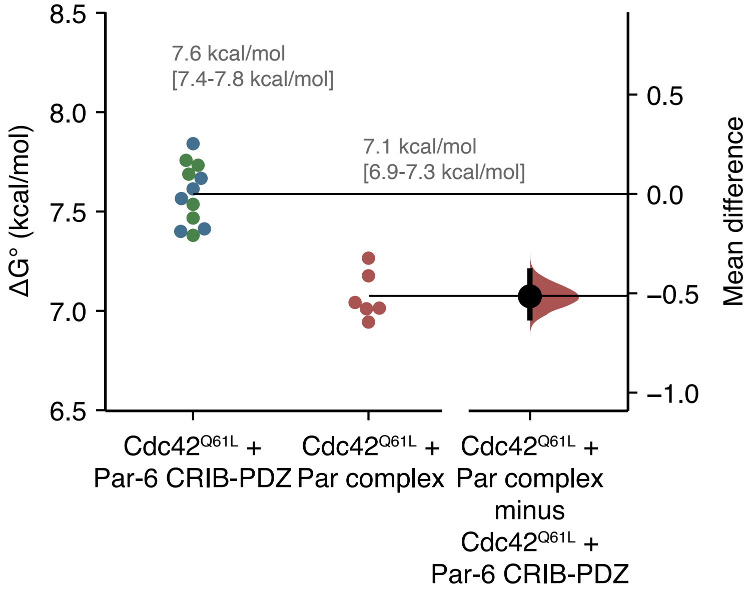

Figure S1 $Cdc42^{Q61L}$ binds with higher affinity to Par-6 CRIB-PDZ than to the Par complex in a supernatant depletion assay. Gardner-Altman estimation plot comparing the binding affinity of $Cdc42^{Q61L}$/Par-6 CRIB-PDZ to the affinity of $Cdc42^{Q61L}$/Par complex measured using a supernatant depletion assay. Each replicate (filled circles) along with the mean and standard devation (gap and bars next to circles) are plotted on the left and the mean difference is plotted on the right as a bootstrap sampling distribution (shaded region) with a 95% confidence interval (black error bar). Different colored circles for $Cdc42^{Q61L}$ + Par-6 represent two assays using either solid phase $Cdc42^{Q61L}$ + soluble Par-6 or soluble $Cdc42^{Q61L}$ + solid phase Par-6.
